# Pathways into and out of temporary disability retirement: an 8-year sequence analysis study in Finland

**DOI:** 10.1093/eurpub/ckaf183

**Published:** 2025-10-10

**Authors:** Anu Polvinen, Aart-Jan Riekhoff, Riku Perhoniemi

**Affiliations:** Finnish Centre for Pensions, Eläketurvakeskus, Helsinki, Finland; Finnish Centre for Pensions, Eläketurvakeskus, Helsinki, Finland; The Social Insurance Institution of Finland, Kela, Helsinki, Finland

## Abstract

Every year, a significant number of people in Finland retire on a disability pension. Half of them are granted a temporary disability pension. Our study examines the different pathways into and out of temporary disability retirement and analyses how individual-level factors are associated with these pathways. We used comprehensive register data on Finns aged 30–58 who received a temporary disability pension in 2018 and followed them for 4 years before and after their pension claim. Sequence analysis in combination with clustering was used to identify typical patterns in and out of temporary disability pension. In addition, we examined the association between individual-level factors and the observed pathways. We identified nine distinct clusters with unique pathways. Two-thirds of the study population belonged to five clusters in which receipt of a disability pension continued for several years. The majority had been employed before receiving the pension. Only 18% belonged to a single cluster characterized by return to work, while persons in the remaining three clusters (20%) ended up in unemployment or unknown labour market states. The different pathways also differed by individual-level factors.

We identified several different pathways into and out of temporary disability pensions. Most of them were characterized by long-term disability retirement rather than return to work. A better understanding of these pathways and associated individual factors is crucial to developing more effective strategies to facilitate return to work and prevent long-term disability.

## Introduction

Increasing employment rates is a key social objective in many Western countries. However, a considerable number of working-age individuals exit the labour market prematurely due to health conditions that lead to work disability. Many countries provide disability benefits for temporary work disability [1]. Efforts are being made to limit the transition to permanent disability benefits, and new benefits are increasingly granted for limited periods only. The aim is to encourage return to work and to support the recovery of working capacity [1, 2]. In Finland, around 20 000 people retire on disability pensions each year. Half of them are granted a temporary disability pension and are expected to return to work. However, re-entry into the workforce is relatively uncommon; many continue to receive disability benefits on a permanent basis [1–5].

To better understand the circumstances leading to temporary disability pension, the labour market transitions that follow, and the relationship between pre- and post-periods, we need more evidence on the various paths and transitions between work, retirement, and unemployment before and after receiving a temporary disability pension. Previous research on those who have transitioned to temporary disability pension has mainly focused on the period after temporary disability retirement and returning to work [4–11]. Relatively little information is available on the phases preceding temporary disability pension [12–14]. It is crucial to identify the early stages that lead to disability and to comprehend how prior events influence the likelihood of returning to work or the persistence of work disability.

Work ability may deteriorate for several years before a disability pension is granted. Many people have long sickness absences before they move into disability pension [15–21], and many disability pension recipients have also experienced spells of unemployment [22, 23]. A previous Finnish study on life course stages prior to temporary disability pension [12] found that just under half of those receiving a temporary disability pension had worked intensively, a quarter had an unstable work history, and just under a third had been unemployed for long periods. The same study found that unemployment prior to disability was particularly high among those claiming a pension for mental disorders other than depression.

Earlier studies have shown that returning to work after receiving temporary disability benefits is relatively rare [1, 4, 5, 7, 9, 10]. In Finland, the proportion of people returning to work has varied between around 10%–25%, depending on the population group and pension type [6]. A Finnish study showed that more than half of temporary disability pension recipients were still receiving a disability pension 4 years after it was granted [5]. Most of them were in permanent disability retirement. The same study [5] also found that a relatively high number of temporary disability pension recipients ended up being unemployed. It is known that for people with work disability, sustained unemployment is relatively common [1]. Many recipients of a temporary disability pension apply for an extension or a permanent disability pension. A large proportion of these applications are rejected, while many applicants are unable to return to work or find suitable employment [24]. Almost half of all rejected applicants are later awarded a disability pension [24, 25].

Many individual-level factors are known to be associated with returning to work or with temporary disability pensions being converted into permanent pensions [4, 5, 10, 11]. Younger people and those with a higher level of education are more likely to return to work. Return to work is more common among individuals receiving a temporary disability pension due to musculoskeletal disease or injury and less common among individuals with mental disorders [4, 5]. People with longer periods of disability are known to be less likely to return to work [7]. In addition, return to work is more common among those who were employed before receiving a temporary disability pension [5, 13]. Men and older people are slightly more likely to move to a permanent disability pension than women and younger people [6]. Previous studies have found that unemployment is associated with poorer mental health and that long periods outside the labour force often lead to low chances of returning to work [18].

It is important to unravel the labour market paths that individuals follow before and after temporary disability pension and to explore how these paths are related. In this study, we follow the employment, unemployment, sickness absence, and retirement paths of individuals who have been assessed as having a temporary reduced capacity to work. Previous studies have mainly focused on return to work after disability pension. The novelty of our study is that it follows individuals for 4 years before and after they start receiving a temporary disability pension, using sequence and cluster analysis to identify different subgroups with unique pathways. Several individual-level factors are known to be associated with disability retirement and return to work, but little is known about how these factors relate to different paths in the longer term. We study how age, gender, education, pension diagnosis, and applications are related to different pathways.

### Work disability benefits in Finland

In Finland, sickness allowance covers short-term work disability due to illness or injury lasting up to 1 year. If work disability persists for more than 1 year, the individual may be eligible to receive a disability pension. If it is assumed that the person will be able to return to work, the disability pension can be granted for a temporary period. When a person with lowered work ability applies for a disability pension, an assessment is also made of whether they would benefit from vocational rehabilitation. This ensures that the applicant has a treatment and rehabilitation plan. There is no maximum duration for a temporary disability pension. If an individual’s ability to work has not been restored by the end of the initial period, the pension may be extended. The total duration of a temporary disability pension can range from a few months to several years [26, 27].

Under the earnings-related pension system, a disability pension is either full or partial. A partial disability pension is granted if the individual’s work ability is reduced by at least 40% for more than 1 year. The purpose of the pension is to allow the person to continue to work while receiving the pension, although there is no obligation to work. A full disability pension is granted if the individual’s work ability is reduced by at least 60%. The assessment is made by an insurance physician and it is based on medical indicators and other factors affecting the applicant’s ability to earn a living [26, 27].

## Methods

We used register data from Statistics Finland and the Finnish Centre for Pensions. Our study included persons aged 30–58 who began receiving a new temporary disability pension in 2018 and who were alive at the end of 2022. Our focus was thus on those working-age individuals who had not previously received a disability pension, who were not able to claim an old-age pension during the follow-up period, and who should have had several years of employment ahead of them before reaching the statutory retirement age. The final study population comprised 7297 individuals.

### Monthly statuses

The register data included detailed information on individuals’ exact start and end dates of employment, unemployment, retirement (both full and partial disability pensions), and spells of sickness benefits. Based on this information, individual monthly statuses were compiled. Individuals were followed for 48 months before and after the onset of a temporary disability pension. This follow-up period ranged from January 2014 to December 2022, depending on the month in which the temporary disability pension was granted in 2018.

Six mutually exclusive states were constructed for each person and for each of the 96 follow-up months. The monthly states were (1) full disability pension, (2) partial disability pension, (3) sickness benefit, (4) unemployment, and (5) employment. A person was classified in each state if they had received the said benefit or been employed for at least 10 days in that month. If none these states could be found for a 1-month unit, the state of that month was recorded as 6) other/unknown. In the case of overlap, the state mentioned earlier in the above list took precedence over the states listed later.

### Background variables

Age was grouped into categories of 30–39, 40–49, and 50–58 years at the beginning of temporary disability pension receipt. Highest educational level was classified as primary school, secondary education, and lower or higher tertiary education.

Diagnoses of diseases leading to temporary disability pension were drawn from the retirement application data. They were categorized according to the International Classification of Diseases (ICD-10) into two diagnostic groups: mental and behavioural disorders (F00-F99) and somatic diseases.

Two additional retirement events were examined as covariates. First, we calculated whether individuals’ applications for full or partial disability pension had been rejected once or more often either before (2014–2018) the temporary disability pension started in 2018 or after receipt of a temporary disability pension (2019–2022). We also examined whether a person’s disability pension had been converted into a permanent pension by the end of 2022.

### Sequence and cluster analysis

The monthly statuses were used to create sequences for the study population so that each individual had a sequence of 96 months before and after the start of their temporary disability pension. We used sequence and cluster analysis to analyse these sequences and to identify typical patterns for these individuals. We applied optimal matching techniques to measure similarities and distances between pairs of sequences. Transition rates between states were used to define substitution costs, which means that a substitution cost between two states is lower if transitions between those states are more common. Transition rate based substitution costs are commonly used in optimal matching as a data-driven way to reduce bias from arbitrary or uniform cost settings. The insertion/deletion (indel) cost was set to the default value 1. The Ward method was used for clustering. Analyses were performed using the TraMineR and WeightedCluster packages [28, 29].

An optimal cluster solution was found based on comparing the performance of various cluster solutions on the Average Silhouette Width (ASW) values for cluster quality [29] and our own assessment of a meaningful distinction between clusters. Although ASW was highest for the two-cluster solution (0.3457), we selected the nine-cluster solution (0.3225) which indicated an almost equally high cluster quality but allowed for more detailed observations in its distinction between clusters. PBC was highest for the nine-cluster solution (0.5856). The AWS indicator for cluster quality is overall relatively low [29, 30]. This may be due to the unusual structure and timing of the states, which include pre-pension periods, the exact moment when all persons start to receive a disability pension, and subsequent periods. Most importantly, the nine-cluster solution provides a meaningful representation of the interlinking of situations prior to receiving a disability pension, the type of pension granted, and the prospects of returning to work. The chosen solution effectively distinguishes between clearly defined cluster identities, each emphasizing different aspects of the examined states.

## Results

**Figure 1.**
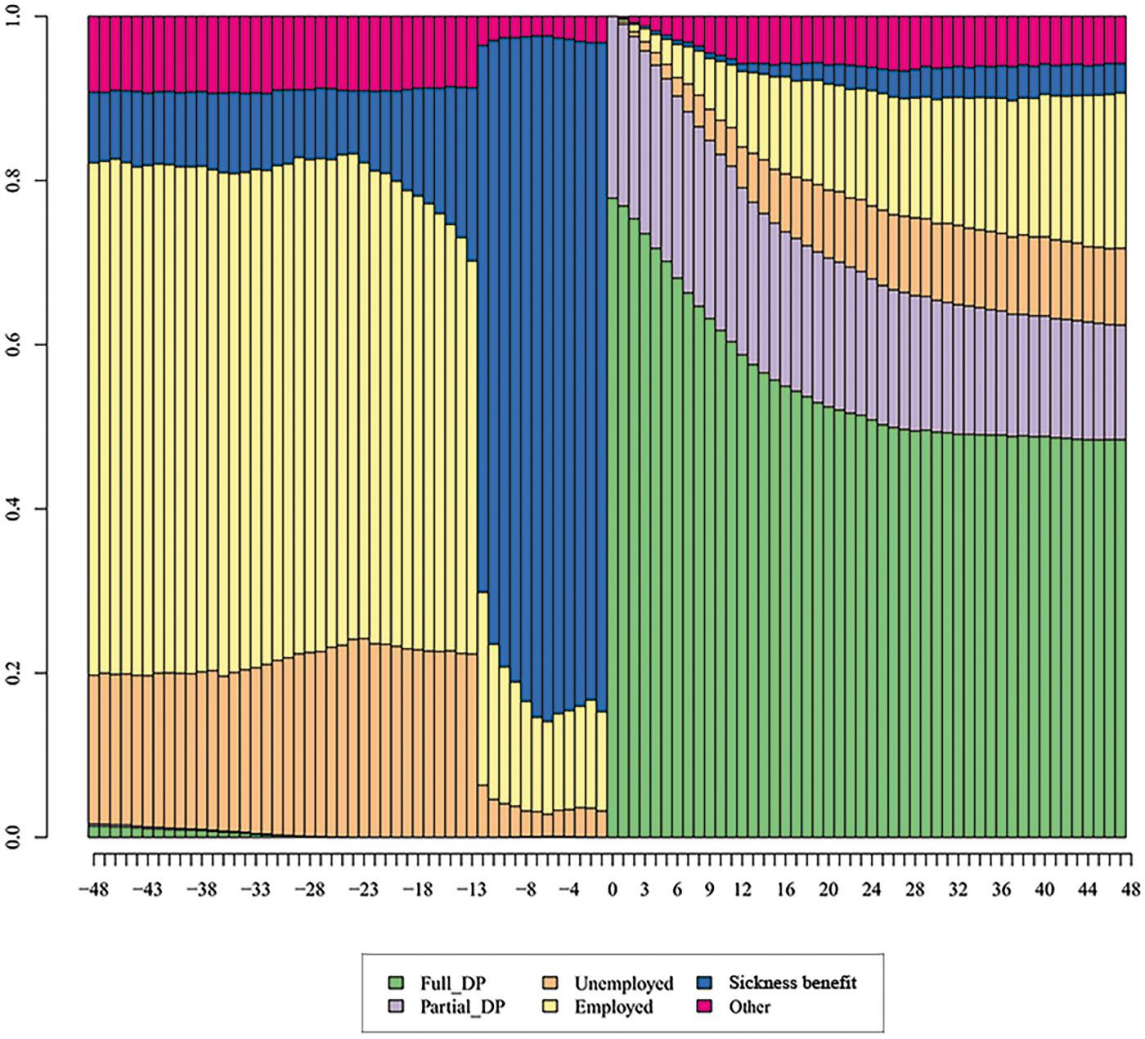
Monthly statuses before and after entering a temporary disability pension (DP).

The distribution plot in Fig. 1 illustrates the frequency of different states over time. The X-axis represents time and the Y-axis represents the share of persons in each state at each point in time. The results showed that 4 years before receiving a temporary disability pension, around 60% of the study population were employed, 20% were unemployed, and 10% were in some other state. In the pre-period, some 10% received sickness benefits each year, rising to 80% 12 months before receipt of a temporary disability pension. This is an expected result because a disability pension is usually preceded by a 12-month period of sickness allowance.

At the start of the temporary disability pension in 2018, 80% of the study population received a full disability pension and 20% a partial disability pension. The proportion of full and partial disability pensioners then began immediately but slowly to decrease over time. Four years later, approximately 50% received a full disability pension and 15% a partial disability pension. One-fifth were employed and almost an equal proportion were either unemployed or in some other state.

**Figure 2.**
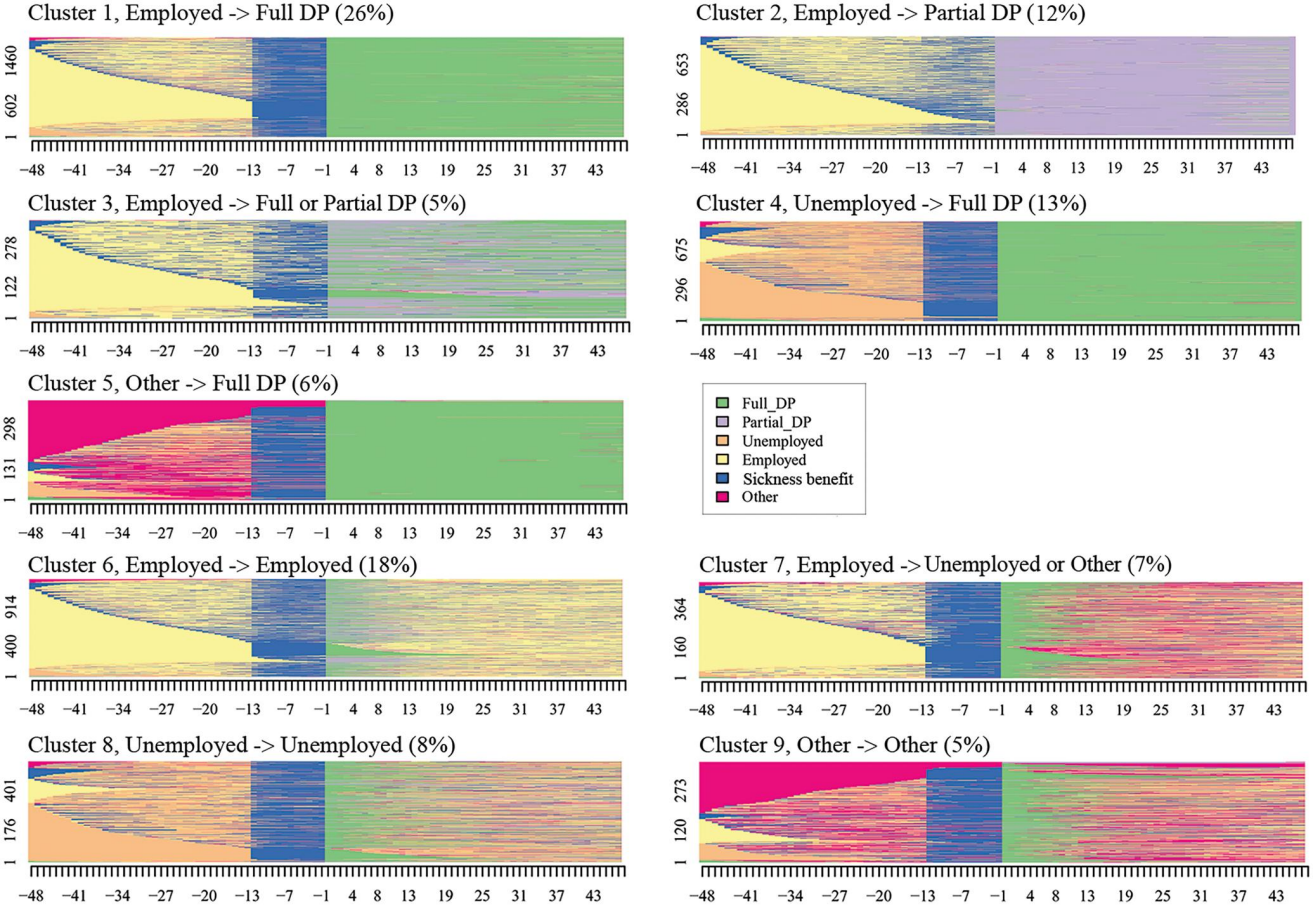
Individual sequences 48 months before and after entering a temporary disability pension (DP) in the nine clusters.

Optimal matching techniques and cluster analysis yielded nine clusters (Fig. 2). In the index plots, each row represents the sequence of statuses of an individual over time. Almost two-thirds or 62% of the study population belonged to clusters where the disability pension seemed to continue for at least 4 years (clusters 1–5). The first cluster included those who were mainly employed before receiving a full disability pension (26%). The second cluster included employed individuals who started receiving a partial disability pension (12%). A further 5% were employed before receiving a temporary disability pension but their pension changed from a partial to full disability pension, or vice versa (cluster 3). Finally, 13% were unemployed (cluster 4) and 6% were in other states (cluster 5) prior to receiving a full disability pension.

Shorter spells of disability pension receipt were recorded for 38% of the study population (clusters 6-9). Around 18% were employed before receiving a temporary disability pension and returned to work after a short period on a disability pension (cluster 6). A further 7% were also employed before a temporary disability retirement but then became unemployed or moved to other states (cluster 7). Roughly, the same proportion or 8% were unemployed prior to disability retirement and mainly ended up back in unemployment (cluster 8). The remaining 5% were in other states and ended up in unemployment or other states after receiving a temporary disability pension (cluster 9).

Table 1 shows the frequencies of background variables and covariates for each cluster. The clusters characterized by transitioning from employment to a disability pension and continued pension receipt for at least 4 years (clusters 1-3) included relatively many individuals aged 50–58 and with a high education, and their pension was typically granted due to somatic diseases. Recipients of a partial disability pension (cluster 2) were older and higher educated than those who transferred to a full disability pension, and the majority of them were women. Those who received a disability pension that continued after unemployment (cluster 4) were more often younger, lower educated, and men than individuals in other clusters. Also, their disability pension was quite often granted due to mental disorders, and their applications for a disability pension had more often been rejected prior to their temporary disability pension compared to those who transitioned from employment to a disability pension. Just over 60% of individuals whose disability pension had lasted for at least 4 years had had their pension converted to a permanent pension, except for those who entered a disability pension from other states (cluster 5).

**Table 1.** Descriptive statistics of clusters, %

	Cluster 1 Employed -> Full DP N = 1921	Cluster 2 Employed -> Partial DP N = 911	Cluster 3 Employed -> Full or Partial DP N = 387	Cluster 4 Unemployed -> Full DP N = 941	Cluster 5 Other -> Full DP N = 415	Cluster 6 Employed -> Employed N = 1277	Cluster 7 Employed -> Unemployed N = 506	Cluster 8 Unemployed -> Unemployed N = 559	Cluster 9 Other -> Other N = 380	All N = 7297
Gender										
Men	45.8	23.9	30.2	48.4	48.7	36.1	51.4	42.2	43.4	41.0
Women	54.2	76.1	69.8	51.6	51.3	63.9	48.6	57.8	56.6	59.0
Age in 2018										
30–39	17.1	8.7	9.3	21.5	38.8	21.2	28.3	22.0	42.4	20.6
40–49	26.6	22.6	23.8	29.5	29.2	35.1	32.6	32.7	28.9	29.0
50–58	56.3	68.7	66.9	49.0	32.0	43.7	39.3	45.3	28.7	50.4
Educational level										
Primary	13.6	5.8	10.9	18.1	18.7	11.8	20.6	22.2	27.1	14.9
Secondary	56.6	52.8	52.2	62.5	53.5	53.5	60.7	58.9	52.8	56.2
Tertiary	29.8	41.2	37.0	19.5	27.9	34.7	18.8	19.0	20.2	28.9
Diagnosis										
Mental	49.0	36.0	36.4	62.0	73.8	34.5	29.8	36.0	46.1	44.8
Somatic	51.0	64.0	63.6	38.0	26.2	65.5	70.2	64.0	54.0	55.2
Rejections of DP claims prior temporary DP										
Yes	14.8	12.2	16.0	33.6	25.6	11.3	19.7	32.4	25.5	19.2
Rejections of DP claims after temporary DP										
Yes	10.1	16.0	22.7	9.0	6.7	22.6	49.6	55.6	40.8	21.2
Transferred to permanent DP										
Yes	62.4	69.7	64.9	65.1	48.7	4.9	5.3	18.6	7.6	42.8

DP, disability pension.

Those who returned to work (cluster 6) were more often women, aged 40–49, and had tertiary education. Their disability pension was often granted due to somatic diseases. They had also received fewer rejections than people in other clusters prior to receiving a temporary disability pension.

Those who ended up in unemployment after initial employment (cluster 7) were often men, relatively young, and low educated. Almost 70% of them had received a temporary disability pension due to somatic diseases. Ending up in unemployment in general (clusters 7-8) was common among those with lower education. In these two clusters, many disability pension claims had been rejected after the start of a temporary disability pension. Those who ended up in other states were often relatively young and less educated, and they also had more rejections of disability pension claims after the onset of their temporary disability pension.

## Discussion

In this study, we used sequence analysis and clustering to identify labour market paths before and after temporary disability retirement. The analysis revealed various pathways into and out of temporary disability retirement, including employment, unemployment, sickness absence, and retirement paths. We found that pathways differed by position of entry (employment, unemployment, or other), type of disability pension (full or partial), and outcome (continued disability, employment, unemployment, or other). Furthermore, our analysis showed that unique combinations of entry, type of disability pension, and outcomes were associated with distinct individual-level characteristics.

Almost two-thirds of people who started receiving a temporary disability pension continued to receive the pension for at least 4 years, and many of them were eventually granted a permanent pension. The majority had been employed before entering a temporary disability pension, while a smaller proportion had been unemployed or otherwise inactive. For some, the disability pension continued as a partial pension, allowing them to work part-time while drawing their pension [31]. Those who transitioned from employment to partial disability pension and who continued to receive the pension were older and more highly educated than individuals in other clusters. According to previous Finnish studies, recipients of a partial disability pension are typically older and more highly educated than those receiving a full disability pension [31, 32]. This may be because individuals with higher education and stable careers are better able to continue working part-time. In contrast, those who moved from unemployment to a disability pension and continued to receive the pension were relatively young and less educated. Previous studies have found a link between unemployment and disability pensions, especially among younger and less educated individuals [22]. Furthermore, younger people are more likely than older individuals to receive a disability pension for mental health reasons [33, 34].

Only a relatively small proportion of individuals returned to work (18%). Our results are in line with previous studies [4–6, 8] in that those who did return to work had predominantly been employed before transitioning to a disability pension. Furthermore, our findings revealed that women, individuals aged 40–49, those with higher education levels, and those whose pensions were granted due to somatic reasons were more likely to return to work. Earlier research [4–6, 8] has similarly highlighted the influence of gender, age, education, and the nature of the disability on the likelihood of returning to work. Individuals aged 40–49 are often in the middle of their careers, with an abundance of skills and knowledge and many active years still ahead of them. Additionally, those with higher education generally have better professional skills and broader expertise, which may improve prospects of re-employment and facilitate adaptation to new job roles. Some of those who had been employed ended up in unemployment after their temporary disability pension. They differed from the group that returned to work in that they were more likely to be men, younger, and less educated.

It seems that individuals who were unemployed or in other unknown states not captured by this study before receiving a temporary disability pension relatively often ended up back in the same state after receiving a temporary pension, or their status fluctuated between unemployment, employment, and other states. The unknown states in this study may include living on other social benefits or savings, but all the same imply being outside paid work and pensions. This result can be seen as reflecting the difficulty of finding work from outside the labour market and with reduced work ability. It is well known that many unemployed people have diminished work ability, making it challenging to return to work [18]. Our results also indicate that many unemployed individuals re-applied for a disability pension but were turned down. This partly reflects that many unemployed individuals may have lowered working ability but not to a sufficient extent to qualify for a permanent disability pension.

### Methodological considerations

While there has been some research to explore return to work after a temporary disability pension, our study provides a more comprehensive view on subgroups on temporal disability pension and sheds new light on the various pathways into and out of temporary disability retirement. The dataset is unique in that it provides monthly statuses for each individual, covering employment, unemployment, sickness benefit days, and retirement on a partial and full disability pension. The follow-up period covers 96 monthly time points for the entire study population. We used sequence and cluster analysis to create different pathways for those who started to receive a temporary disability pension in 2018. In a model, individuals had the same time-point when their status changed from employment, unemployment, sickness benefits, or other states to either full or partial disability pension. Our cluster solution meaningfully distinguishes between different trajectories through employment, unemployment, sickness absence, other state, and disability pension. This indicates that our analysis effectively captures the transitions within the study population, providing valuable insights for further research and policymaking.

## Conclusions

Recipients of a temporary disability pension are likely to transition to a permanent disability pension, with only a small proportion returning to work. This suggests that the treatment or rehabilitation plans required for recipients of a temporary disability pension are not particularly effective. By better understanding the different pathways into and out of temporary disability retirement and more accurately identifying the groups at higher risk of transitioning to permanent disability, we can eventually offer more personalized support through tailored rehabilitation programmes or redesigned job tasks, for example. It is crucial to develop more effective strategies that can help facilitate the return to work and mitigate long-term disability.

## Data Availability

The authors used individual-level register data from Statistics Finland and the Finnish Centre for Pensions. Due to legal restrictions and data protection regulations, the authors do not have permission to make sensitive personal data available.
